# Deep Learning-Based Image Classification of Pupae from 11 Lepidoptera Pest Species

**DOI:** 10.3390/insects17030327

**Published:** 2026-03-17

**Authors:** Zitao Li, Xuankun Li

**Affiliations:** 1 State Key Laboratory of Agricultural and Forestry Biosecurity, College of Plant Protection, China Agricultural University, Beijing 100193, China; lizitao2005@gmail.com; 2 Department of Entomology, College of Plant Protection, China Agricultural University, Beijing 100193, China

**Keywords:** deep learning, image classification, lepidopteran pests, pupal identification, agricultural entomology

## Abstract

The morphological identification of lepidopteran pest pupae has long been a difficult task. To explore automated solutions, this study established a standardized, multi-angle image dataset of pupae from 11 economically important lepidopteran pests. We then systematically evaluated six deep learning models, including both convolutional neural networks and Transformer architectures. The results show that all models successfully learned to distinguish the vast majority of species, with Vit-Small achieving the highest accuracy (98.71 ± 0.16%) and the highest F1-score (98.69 ± 0.20%). This confirms that pupal morphology provides sufficient discriminative visual information to support highly accurate automated identification. However, all models exhibited consistent, minor confusion among *Helicoverpa armigera*, *Mythimna separata* and *Spodoptera exigua*. Analysis revealed these errors originated from specific viewing angles of a limited number of specimens, underscoring the value of the multi-angle imaging protocol used in this study. This study transforms pupal identification from a traditional taxonomic difficulty into a solvable computer vision task, providing a dataset, methodological benchmarks, and a feasibility validation for developing image-based tools for pupal-stage pest surveillance.

## Simple Summary

Traditionally, distinguishing pupae of lepidopteran pests has been challenging due to their subtle morphological differences. To overcome this, we constructed a multi-angle image dataset of pupae from 11 economically important moth pests and tested six state-of-the-art deep learning models for automated identification. The models successfully learned to identify the species with high accuracy—the best reaching over 98% accuracy—confirming that pupal images contain enough visual information for reliable machine-based classification. This work demonstrates a practical path toward developing rapid, image-based tools for pupal pest monitoring in the field, which could significantly improve early detection and management in agriculture.

## 1. Introduction

Lepidopteran pests pose a severe and persistent threat to global agriculture and forestry, characterized by high reproductive potential, migratory behavior, and the rapid development of pesticide resistance [1,2,3]. Many significant agricultural pests belong to the families Noctuidae, Crambidae, and Erebidae, encompassing a wide range of species that attack various crops through diverse feeding behaviors [4,5]. For instance, the Old World cotton bollworm *Helicoverpa armigera*, a highly polyphagous species native to Asia, Europe, Africa, and Australasia, was recently confirmed to have successfully invaded South America, causing significant damage to maize and cotton in Brazil [6]. Similarly, the fall armyworm *Spodoptera frugiperda*, after its initial detection in Africa in 2016, rapidly spread across the continent and was reported in southern India by 2018, where it inflicted severe injury to maize crops, with incidence rates reaching as high as 62.5% in some districts [7]. Accurate and timely species identification is critical for effective integrated pest management (IPM), enabling targeted surveillance and control [8,9]. For Lepidoptera, traditional taxonomy primarily relies on adult morphology (e.g., wing venation, genitalia) and, to a lesser extent, larval characteristics [10,11,12]. In contrast, the pupal stage presents a distinct and long-standing challenge for morphological identification [13,14]. Pupae often exhibit fewer diagnostic characters, and their taxonomy is consequently understudied, rarely being used for identification purposes in field settings. This gap is particularly critical, as the pupal stage is a key overwintering or oversummering form for many species, making its detection and identification vital for predicting population dynamics and outbreak risks [15].

Recent advances in deep learning, particularly convolutional neural networks (CNNs), have demonstrated remarkable success in automating the image-based identification of insects across diverse taxa, showcasing their ability to learn discriminative morphological features directly from images [16,17,18,19]. The underlying principle of CNNs, that they can extract subtle, consistent visual patterns, makes them a promising candidate for overcoming the taxonomic challenges posed by pupae. However, this potential remains largely untested and unquantified: current studies have mostly used images from adult insect stages (e.g., Ling et al., 2023 [20], Shirali et al., 2024 [21] and Zhao et al., 2023 [22]) or immature insect stages (e.g., Simović et al., 2024 [23], Kodors et al., 2025 [24] and Xu et al., 2022 [25]). Moreover, the few existing applications targeting pupae have focused on sex and development stage classification and silkworm breeding strain identification, rather than multi-species discrimination [26,27]. The development of robust, deep learning-based tools is fundamentally constrained by the availability of high-quality pupal image datasets.

**Figure 1:**
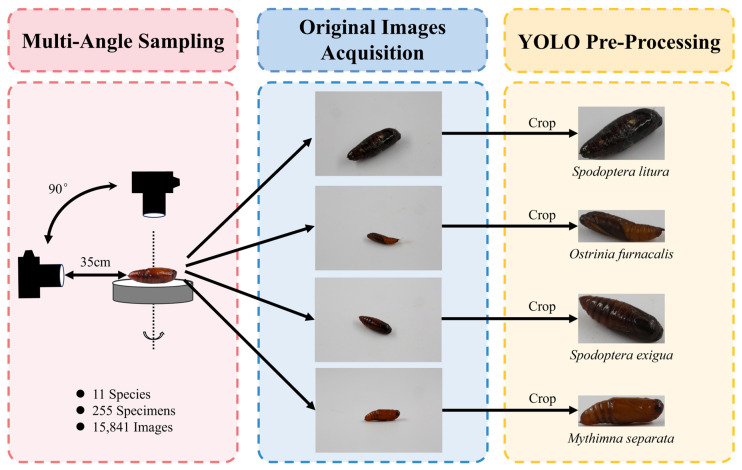
Data collection flowchart of pupae of 11 Lepidopteran pest species.

To this end, we conducted a comprehensive case study focusing on 11 economically significant Lepidopteran pest species. We present the first multi-species identification of Lepidoptera at the pupal stage. We first constructed a standardized image dataset of their pupae, in which each specimen was systematically imaged from multiple angles to capture comprehensive three-dimensional morphological information (Figure 1). Using this dataset, we then rigorously trained and evaluated a suite of state-of-the-art deep learning models to establish baseline performance for pupal identification and to identify which architectural approaches are most effective. By providing both the dataset and a fully documented, reproducible analysis pipeline, this work offers a foundational reference point for future research. The methodology and standards employed here are designed to be extensible, inviting contributions from entomologists and taxonomists to incorporate additional species. Our goal is to initiate a community-driven effort that, through accumulated data and shared models, can eventually deliver robust, field-deployable tools for pupal monitoring. The successful development of such tools would directly address a critical gap in surveillance, enabling the earlier detection of pest populations and thereby supporting more proactive and precise agricultural pest management.

## 2. Materials and Methods

### 2.1. Specimen Collection

The pupal specimens used in this study comprised 11 species of lepidopteran pests spanning three families (Crambidae, Erebidae, and Noctuidae) and ten genera (Table 1). All specimens were sourced from established, laboratory-reared colonies to ensure taxonomic reliability (Table 1). All specimens were obtained directly at the pupal stage for imaging, and they were clean upon delivery, with an approximately 1:1 sex ratio. The pupae used were collected with approximately five days of age difference during the middle-to-late phase of pupal development; therefore, they exhibited some natural variation in age and coloration. Voucher specimens (adults reared from the same cohorts) have been mounted and deposited in the Entomological Museum of China Agriculture University (CAU). Species identifications were further confirmed by examining morphological characters of reared adults [28,29].

### 2.2. Images Acquisition

A standardized imaging protocol was employed to capture comprehensive morphological data. Individual pupae were placed on a rotating platform inside a light-diffusing softbox illuminated by uniform LED light. Images were captured using a digital single-lens reflex camera (Canon EOS 80D or Canon EOS 5D Mark III, Canon Inc., Tokyo, Japan) equipped with a macro lens (EF 100 mm f/2.8 L Macro IS USM, Canon Inc., Tokyo, Japan). To obtain detailed three-dimensional information, each specimen was systematically photographed at multiple angles from 0° to 90° relative to the horizontal plane (Figure 1). The numbers of specimens and acquired images are summarized in Table 1. All images were saved in JPG format and named systematically using the convention: “SpeciesID_IndividualID_SequentialID.JPG”.

### 2.3. Data Pre-Processing

To standardize inputs, we implemented a two-stage preprocessing pipeline following [27]. First, an object detection model was trained on a manually annotated subset of 30 images per species to localize pupae within each frame [30,31,32]. This model was then used with X-AnyLabeling (version 3.3.1) [33] to automatically localize and crop the primary sections of the pupae from all subsequent images. This process yielded a 2.42 GB dataset with a total of 15,349 cropped images (Table 2 and Table S1), which formed the final dataset used for all subsequent classification tasks.

All cropped images were then resized and normalized for deep learning model training. Images were randomly resized and cropped to a uniform dimension of 224 × 224 pixels. Pixel values were normalized using the mean ([0.485, 0.456, 0.406]) and standard deviation ([0.229, 0.224, 0.226]) derived from the ImageNet dataset [34]. To improve model generalizability and robustness, we applied a standard set of online data augmentations during training, including random horizontal flipping (*p* = 0.5), random rotation within a range of ±15 degrees, and random adjustments to color jittering (brightness, contrast, and saturation, each with a factor of 0.2) [35]. For the validation and testing phases, the augmentation steps were disabled, and images were resized directly to 224 × 224 pixels.

The processed image dataset was then partitioned at the specimen level into training, validation, and test subsets in an approximate 8:1:1 ratio (Table 2). This strict partitioning guaranteed that all images from a single physical specimen were contained within one subset, thereby preventing data leakage during model training and evaluation. Data augmentations are applied on-the-fly during each training epoch. Consequently, although the training set consists of 11,970 base images (Table S1), the model processes an online augmented version of each image in every epoch. Over the 30 training epochs, this strategy effectively exposes the model to a wide distribution of variants, with an upper bound of 30 × 11,970 = 359,100 distinct augmented views. Across the entire training process, the model benefits from a wide distribution of augmented views.

### 2.4. Model Training

To benchmark the performance of different deep learning architectures on the pupal image classification task, we selected and trained six representative models. This included four convolutional neural networks (CNNs): EfficientNet-B0 [36], MobileNet-v2 [37], ResNet-50 [38], and ConvNeXt-B [39]; and two Transformer-based models: Vision Transformer Small [40] and Swin Transformer Tiny [41]. This selection aimed to compare classical and modern CNN designs with the emerging Transformer paradigm on pupa recognition tasks.

To ensure a fair and rigorous evaluation, we implemented a standardized training pipeline. We repeated each experiment five times using random seeds 40 to 44 and report the final results as mean ± standard error to ensure the robustness of the results [42,43]. All models were initialized with weights pretrained on the ImageNet-1K dataset. They were trained using the AdamW optimizer with a weight decay of 0.05 [44] and a cosine annealing learning rate scheduler [45]. To ensure comparability across different architectures, consistent hyperparameters were applied to all architectures: a batch size of 128, an initial learning rate of 5 × 10^−5^, and a total of 30 training epochs. Optimization was performed using the standard cross-entropy loss function. All training and evaluations were conducted on Intel Xeon Ice Lake 8358 CPU@2.6GHz (Santa Clara, CA, USA) and NVIDIA HGX A800 80G GPUs (Santa Clara, CA, USA) using PyTorch 2.4.1 on CAU high-performance computing (HPC) platform.

### 2.5. Model Evaluation

Model performance was evaluated on the held-out test set. Quantitative evaluation was based on standard multi-class classification metrics: accuracy, macro-averaged precision, macro-averaged recall, and macro-averaged F1-score.

In addition to overall metrics, we evaluated the practical reliability of the identification system for each species by calculating two taxon-specific error rates: the False Discovery Rate (FDR) and the False Negative Rate (FNR) [46,47]. The FDR, defined as FP/(TP + FP), quantifies the proportion of predictions for a given species that are incorrect (i.e., false alarms). Conversely, the FNR, defined as FN/(TP + FN), measures the proportion of true instances of a species that are missed by the classifier (i.e., missed detections). This analysis was performed using the predictions from the best-performing model (ConvNeXt-B) on the held-out test set.

### 2.6. Visualization and Interpretation

To interpret the model’s decision-making process and assess the biological relevance of learned features, we employed Gradient-weighted Class Activation Mapping (Grad-CAM) [48]. This technique generates class-discriminative heatmaps by backpropagating the gradients of a target class through a model’s final feature-generating layers [49,50]. The method was adapted for different architectures: for convolutional neural networks (CNNs), we applied Grad-CAM to the final convolutional layer; for Transformer-based models, which lack convolutional layers, we computed gradients and activations from the projection layers within the final attention blocks, as these layers integrate global contextual information crucial for the model’s predictions.

## 3. Results & Discussion

### 3.1. Classification Performance

The classification performance of six deep learning architectures on the pupal image test set is summarized in Table 3. Vit-Small emerged as the top performer, followed by ConvNeXt-B. Vit-Small achieved the highest accuracy (98.71 ± 0.16%), precision (98.83 ± 0.14%), recall (98.61 ± 0.25%) and F1-score (98.69 ± 0.20%), indicating exceptional predictive consistency. Its success, particularly in precision, suggests that the global self-attention mechanism inherent to Vision Transformers may be highly effective at integrating contextual information across the entire pupal image, thereby reducing false positives in this fine-grained classification task [40,51,52]. Conversely, ConvNeXt-B attained the second highest four metrices, demonstrating a superior and balanced capability in detecting positive samples. As a modern CNN that incorporates Transformer-inspired design elements (e.g., larger kernel sizes), its leading F1-score underscores how architectural refinements within the convolutional paradigm can achieve robust performance, making it a strong candidate for applications requiring a reliable balance between detection rate and prediction certainty.

**Figure 2:**
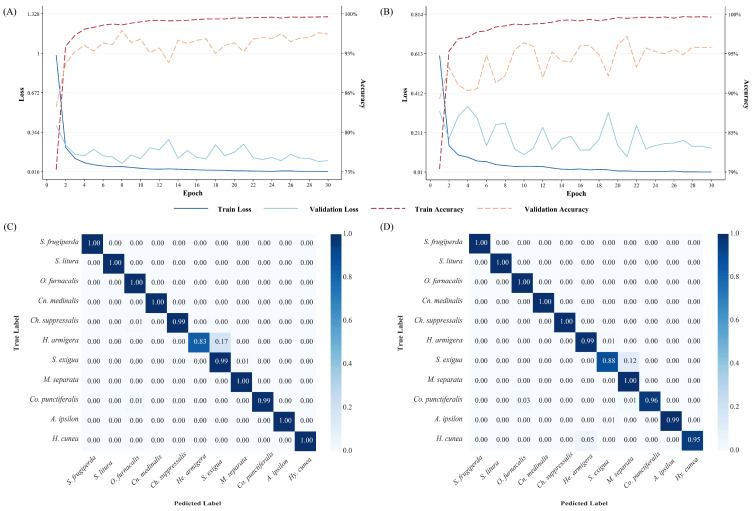
Accuracy and loss curves and of normalized confusion matrixes of two best performed architectures. (**A**,**C**) ConvNeXt-B; (**B**,**D**) Vit-Small.

A detailed analysis of training curves and confusion matrices (Figure 2 and Figures S1–S4) revealed consistently high overall identification rates across the 11 moth species. Similar to other studies, models based on CNN and Transformer architectures have achieved good performance in image classification tasks [18,19]. However, a small, persistent cluster of misclassifications was observed among three noctuid species: *Helicoverpa armigera*, *Spodoptera exigua* and *Mythimna separata*. To investigate these errors, we calculated the False Discovery Rate (FDR) and False Negative Rate (FNR) for them using three high-performing models (ConvNeXt-B, Swin-Tiny and Vit-Small). As shown in Table 4, *He. armigera* exhibited a relatively high FNR, indicating that true instances of this species were often missed by the classifier and misidentified as another (often *S. exigua*). Conversely, *M. separata* showed a notably high FDR, meaning it was frequently predicted when the true species was something else (often *He. armigera*). *S. exigua* displayed elevated levels in both error metrics in ResNet-50 model. This specific confusion pattern merits further investigation to determine if it stems from overlapping visual features, limitations in specific viewing angles, or other dataset characteristics.

### 3.2. Interpretability via Grad-CAM

**Figure 3:**
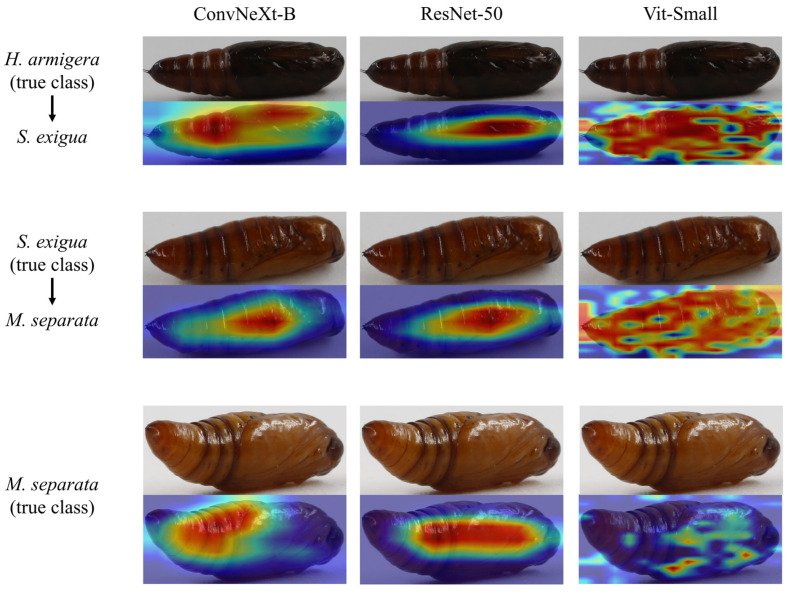
Grad-CAM visualizations for moth pupa images of *He. armigera* (confused with *S. exigua*) and *S. exigua* (confused with *M. separata*). The activation maps highlight the regions attended by different models during classification, with color intensity indicating the degree of model focus: red regions correspond to higher attention and blue regions to lower attention.

We applied Grad-CAM to understand the basis of model predictions and to investigate the persistent misclassifications identified in Section 3.1. The visualizations revealed a notable difference in how architectures attend to image features (Figure 3). CNN-based models (ResNet-50, ConvNeXt-B) consistently attend to coherent regions of the pupal body with little background information, aligning with both effective feature extraction for classification and the morphological traits used in insect taxonomy (e.g., abdominal spines and spiracles). In contrast, the Transformer-based model (Vit-Small) occasionally concentrated on background details or on overly localized patches in some samples, though it still achieved high overall accuracy.

All three families were precisely distinguished. Across architectures, consistent misidentifications occur primarily among noctuid species: *He. armigera* confused with *S. exigua*, and *S. exigua* confused with *M. separata*. Morphologically, these species can be distinguished by several key characteristics. The pupae of *S. exigua* exhibit a slightly straight anterior margin of the mesonotum and a short, stout cremaster bearing a pair of widely separated terminal spines [53,54]. In contrast, *He. armigera* pupae also possess separated terminal spines but are characterized by sparse, semicircular punctures on the dorsal side of abdominal segments 5–7 [53,55]. Meanwhile, *M. separata* pupae display a row of large, irregularly wavy notches along the anterior margin of abdominal segments 5–7 dorsally, along with a cremaster featuring a pair of thicker dark brown spines flanked by smaller yellowish hook-like spines [53].

In this study, most misclassified images originated from the same set of specimens (Specimen ID: 1108_025 and 1110_023) and in certain angles (Figure 3). Notably, these errors were consistent across all six model architectures, despite the confirmed taxonomic accuracy of all specimens via adult rearing. This indicates that the difficulty is inherent to the visual information contained in these specific angles. For these challenging cases, the models’ salient regions were often less distinct or failed to converge on the clearest diagnostic features.

### 3.3. Discussion of Model Interpretability and Taxonomic Alignment

The observed pattern of errors can be interpreted through the fundamental difference between deep learning and traditional taxonomic reasoning. In deep learning models, the morphological features prioritized for classification often differ from those used by human taxonomists, particularly in fine-grained image recognition tasks [56,57]. In our pipeline, all images were resized to a uniform resolution (224 × 224 pixels), rendering the model scale-invariant and eliminating any potential influence of absolute pupal size on inference. This design choice is intentional and practically advantageous: it removes the need for users to place a ruler next to the specimen when imaging or to measure and report size to the model, thereby facilitating potential field applications. Moreover, while pupae from laboratory-reared colonies are relatively uniform in size, body size can vary considerably under field conditions due to resource availability, further justifying the exclusion of absolute size as a reliable diagnostic feature.

Taxonomists typically rely on discrete diagnostic characters, where the value of a feature increases if it does not overlap with traits of other species. In contrast, deep learning approaches assign weights to all visible features and make decisions through an integrated, comprehensive assessment [40,58,59]. This distinction parallels how experienced taxonomists often recognize specimens instantly by overall gestalt rather than consciously checking each diagnostic character—the model develops similar intuitive familiarity through extensive training. Using Grad-CAM visualization, we found that the model consistently attends to taxonomically relevant regions including anterior segments, posterior segments (where the cremaster is located), and intersegmental boundaries (Figure 3). While this does not constitute formal integration with traditional taxonomic morphology, it demonstrates that the model learns to focus on biologically meaningful areas rather than background or artifacts, confirming that deep learning captures genuine morphological signals sufficient to support the feasibility of our approach.

For specific images and viewing angles, the visual expression of key characters may have been ambiguous or obscured, reducing the models’ ability to leverage them for a confident distinction. The divergence between a model’s weighted, holistic assessment of all pixels and a taxonomist’s reliance on discrete, non-overlapping characters may explain why specimens that are morphologically distinct in principle can appear visually similar from certain perspectives.

This pattern of misclassification underscores a practical challenge with direct implications for real-world deployment: the visual ambiguity of key diagnostic characters from certain perspectives represents an inherent limitation of relying on single-view inputs. While the CNN demonstrated marginally better utilization of morphological features in its attention maps, both architectures were susceptible to viewpoint-induced ambiguity. In such contexts, capturing and aggregating information from multiple angles could substantially reduce the risk of misidentification caused by any single unfavorable viewpoint.

As demonstrated in Wang et al. 2026 [30], models trained exclusively on standard specimen images showed substantially reduced accuracy when tested on images with diverse backgrounds, lighting conditions, and viewing angles. Their study also revealed that incorporating multi-angle images during training can significantly improve model generalization ability. Following this best practice, our current system is primarily designed for laboratory-controlled imaging conditions while demonstrating the viability of deep learning for lepidopteran pupal identification. For future research, we recommend incorporating field images with diverse backgrounds and lighting conditions, adopting the multi-angle imaging protocol used in this study wherever possible, capturing multiple images of each pupa from different angles to compensate for character ambiguity in any single view, and systematically sampling specimens across complete developmental time series. These approaches would increase model robustness and enable the development of more comprehensive models for real-world applications.

## 4. Conclusions

This study demonstrates that deep learning can reliably identify lepidopteran pests from their pupal stage, a task traditionally challenging even for taxonomists. We generated a standardized, multi-angle image dataset of 11 economically important species and used it to benchmark six modern deep learning models (including both convolutional and Transformer-based paradigms), which achieved high classification accuracy. This validates the pupal stage as a viable source of discriminative features for automated identification, with Grad-CAM analysis revealing that models focus on taxonomically relevant characters. This work provides a practical foundation and an extensible methodological pipeline for building field-deployable tools aimed at the non-destructive, early detection of pests in their pupal stage. The observed error pattern underscores the value of the multi-angle imaging protocol used in this study and suggests that for maximum reliability, future systems may need to integrate information from multiple views to compensate for character ambiguity in any single view. Importantly, for such tools to be truly useful under field conditions, future datasets must prioritize accurate species identification, as mislabeled images remain a critical bottleneck in developing robust models for real-world applications. In summary, this research reframes pupal identification from a taxonomic challenge into a tractable computer vision task. By publicly releasing the dataset and a reproducible analysis framework, we aim to catalyze further community-driven development, ultimately leading to simpler and more accessible tools that enable non-specialists to reliably distinguish pupae of pest moths and supporting more proactive and precise integrated pest management.

## Figures and Tables

**Table 1:** Pupae of 11 Lepidopteran pest species collected for deep learning model training.

Family	Species	Common Name	Specimens	Source
Crambidae	*Chilo suppressalis*	rice stem borer	26	Jiyuan Baiyun Industrial Co., Jiyuan, China
Crambidae	*Cnaphalocrocis medinalis*	rice leaf roller	25	Zhejiang Normal University, Jinhua, China
Crambidae	*Conogethes punctiferalis*	peach borer	21	Jiyuan Baiyun Industrial Co., Jinhua, China
Crambidae	*Ostrinia furnacalis*	Asian corn borer	24	Chinese Academy of Agricultural Sciences, Beijing, China
Erebidae	*Hyphantria cunea*	fall webworm	2	Chinese Academy of Forestry, Beijing, China
Noctuidae	*Agrotis ipsilon*	black cutworm	26	Jiyuan Baiyun Industrial Co., Jinhua, China
Noctuidae	*Helicoverpa armigera*	cotton bollworm	25	Jiyuan Baiyun Industrial Co., Jinhua, China
Noctuidae	*Mythimna separata*	oriental armyworm	26	Jiyuan Baiyun Industrial Co., Jinhua, China
Noctuidae	*Spodoptera exigua*	beet armyworm	25	Jiyuan Baiyun Industrial Co., Jinhua, China
Noctuidae	*Spodoptera frugiperda*	fall armyworm	30	China Agricultural University, Beijing, China
Noctuidae	*Spodoptera litura*	tobacco cutworm	25	Jiyuan Baiyun Industrial Co., Jinhua, China

**Table 2:** Composition of the pupal dataset used for image classification training. The training, validation, and test sets were approximately split in an 8:1:1 ratio.

Species	Images	Train	Val	Test
*A. ipsilon*	2167	1774	210	183
*Ch. suppressalis*	1518	1175	175	168
*Cn. medinalis*	1711	1312	197	202
*Co. punctiferalis*	1469	1137	166	166
*He. armigera*	1352	1036	161	155
*Hy. cunea*	171	136	16	19
*M. separata*	1854	1525	163	166
*O. furnacalis*	1219	954	141	124
*S. exigua*	1171	832	177	162
*S. frugiperda*	1359	1060	146	153
*S. litura*	1358	1029	167	162

**Table 3:** Comparative classification performance of six deep learning architectures. The table presents the accuracy, macro-averaged precision, recall, and F1-score achieved by each model on the test set (Mean ± SEM). The highest value for each metric is highlighted in **bold**. Raw data for calculation is deposited in Table S2.

Architecture	Accuracy	Precision	Recall	F1-Score
ConvNeXt-B	98.48 ± 0.22%	98.66 ± 0.18%	98.60 ± 0.19%	98.58 ± 0.02%
EfficientNet-B0	95.55 ± 0.33%	96.18 ± 0.21%	95.89 ± 0.29%	95.72 ± 0.27%
MobileNet-v2	92.84 ± 0.42%	93.36 ± 0.24%	93.23 ± 0.40%	92.76 ± 0.33%
ResNet-50	97.33 ± 0.18%	97.63 ± 0.15%	97.53 ± 0.16%	97.48 ± 0.17%
Swin-Tiny	97.78 ± 0.39%	98.00 ± 0.35%	97.97 ± 0.36%	97.92 ± 0.37%
**Vit-Small**	**98** **.** **71 ± 0** **.** **16** **%**	**9** **8** **.** **83 ± 0** **.** **14** **%**	**98** **.** **61 ± 0** **.** **25** **%**	**98** **.** **69 ± 0.** **20%**

**Table 4:** Comparison of False Discovery Rate (FDR) and False Negative Rate (FNR) of CNN and Transformer architectures: CovNeXt-B (C), Swin-Tiny (S) and Vit-Small (V). Evaluation was performed using a random seed of 42. Raw data for calculation is deposited in Table S3.

Species	FDR(C)	FNR(C)	FDR(S)	FNR(S)	FDR(V)	FNR(V)
*He. armigera*	0%	8.68 ± 2.72%	0%	10.85 ± 1.48%	0.37 ± 0.15%	4.22 ± 1.5%
*S. exigua*	8.5 ± 2.3%	5.31 ± 1.71%	11.24 ± 1.74%	8.99 ± 2.99%	4.29 ± 1.46%	7.04 ± 1.75%
*M. separata*	5.21 ± 1.62%	0%	9.8 ± 3.42%	0%	6.81 ± 1.72%	0.13 ± 0.13%

## Data Availability

All code and dataset could be found at Github (https://github.com/lizitao2005/MothPupaClassification (accessed on 1 February 2026)) and Zenedo (https://zenodo.org/records/17601422 (accessed on 14 March 2026)).

## Supplementary Materials

The following supporting information can be downloaded at: https://www.mdpi.com/article/10.3390/insects17030327/s1, Figure S1: Accuracy and loss curves and of normalized confusion matrixes of EfficientNet-B0.; Figure S2: Accuracy and loss curves and of normalized confusion matrixes of MobileNet-v2.; Figure S3: Accuracy and loss curves and of normalized confusion matrixes of ResNet-50.; Figure S4: Accuracy and loss curves and of normalized confusion matrixes of Swin-Tiny.; Table S1: Details of pupa dataset for model training.; Table S2: Raw data of four evaluation metrics for six CNN and Transformer models on the pupa dataset (seeds 40–44).; Table S3: Raw data of FDR and FNR for ConvNeXt-B, Swin-Tiny and Vit-Small on the pupa dataset (seeds 40–44).
